# Hyper-Frequency Network Topology Changes During Choral Singing

**DOI:** 10.3389/fphys.2019.00207

**Published:** 2019-03-06

**Authors:** Viktor Müller, Julia A. M. Delius, Ulman Lindenberger

**Affiliations:** ^1^Center for Lifespan Psychology, Max Planck Institute for Human Development, Berlin, Germany; ^2^Max Planck UCL Centre for Computational Psychiatry and Ageing Research, London, United Kingdom; ^3^Max Planck UCL Centre for Computational Psychiatry and Ageing Research, Berlin, Germany

**Keywords:** within-frequency coupling, cross-frequency coupling, cardiac and respiratory autonomic responses, interpersonal action coordination, heart rate variability, graph-theoretic approach, social networks

## Abstract

Choral singing requires the coordination of physiological subsystems within and across individuals. Previously, we suggested that the choir functions as a superordinate system that imposes boundary conditions on the dynamic features of the individual singers and found reliable differences in the network topography by analyzing within- and cross-frequency couplings (WFC and CFC, respectively). Here, we further refine our analyses to investigate hyper-frequency network (HFN) topology structures (i.e., the layout or arrangement of connections) using a graph-theoretical approach. In a sample of eleven singers and one conductor engaged in choral singing (aged between 23 and 56 years, and including five men and seven women), we calculated phase coupling (WFC and CFC) between respiratory, cardiac, and vocalizing subsystems across ten frequencies of interest. All these couplings were used for construction of HFN with nodes being a combination of frequency components and subsystems across choir participants. With regard to the network topology measures, we found that clustering coefficients (*CC*s) as well as local and global efficiency were highest and characteristic path lengths, correspondingly, were shortest when the choir sang a canon in parts as compared to singing it in unison. Furthermore, these metrics revealed a significant relationship to individual heart rate, as an indicator of arousal, and to an index of heart rate variability indicated by the *LF/HF* ratio (low and high frequency, respectively), and reflecting the balance between sympathetic and parasympathetic activity. In addition, we found that the *CC* and local efficiency for groups singing the same canon part were higher than for groups of singers constructed randomly *post hoc*, indicating stronger neighbor–neighbor connections in the former. We conclude that network topology dynamics are a crucial determinant of group behavior and may represent a potent biomarker for social interaction.

## Introduction

In 1619, Johannes Kepler noted, “A collection of individual notes does not in itself form a melody; the melody comes only when we produce a particular arrangement of the individual notes. Harmony, therefore, is produced by the mental activity which recognizes the proportional relationships between the multiplicity of individual parts” (cited in Emerson, 1939, p. 182). In our previous work (Müller et al., 2018a), we suggested that a choir functions as a superorganism or a superordinate system that imposes boundary conditions on its constituents (Wheeler, 1926; Emerson, 1939; Detrain and Deneubourg, 2006). Superordinate systems enclose all other systems in a given time and space, and are characterized through multilevel dynamics as well as upward and downward causation (Noble, 2012). Such superordinate systems are “true emergents, in which whole organisms function as the interacting determining parts” (Wheeler, 1926, p. 435). Interactions among the parts of the system or superordinate system can best be represented in terms of a GTA, whereby graph or network topology measures, such as the *CC* and *CPL* as well as local and global efficiency (*E_local_* and *E_global_*, respectively), are able to provide crucial information about systemic interactions. Using a network construction method based on within- and cross-frequency coupling (WFC and CFC, respectively) of respiratory, cardiac, and acoustic signals, we have recently shown that WFC is enhanced when a choir sings a canon in unison, while CFC is increased when singing the canon in parts (Müller et al., 2018a). In the present work, we aimed to investigate the network topology of a choir HFN using GTA and to explore whether canon groups (choir members singing the same part) are different in their topology as compared to randomly constructed groups of choir members. Furthermore, we wanted to examine whether canon groups, as particular constituents of the superordinate system, exhibit the same or a similar network topology as the superordinate system (i.e., the choir) itself and how this topology changes under different choral conditions.

In the case of choir singing, HFN is a dynamic structure comprising information about phase coupling within and between the frequencies, subsystems, and choir participants. It signifies that subsystems of the choir participants oscillating at different frequencies interact with each other at the same, or at different frequencies. CFC indicates situations where *n* periods of one oscillation are equal to *m* periods of another. In this way, the HFN integrates all the information within a common network that can be analyzed or represented by network topology. The most common topology measures are *CC*, *CPL*, *E_local_*, and *E_global_*, two of them (i.e., *CC* and *E_local_*) being measures of network segregation and the other two (i.e., *CPL* and *E_global_*) being measures of network integration (Watts and Strogatz, 1998; Latora and Marchiori, 2001).

Finally, we aimed to explore how HFN topology is related to physiological indices of the choir members, namely heart rate (*HR*) and heart rate variability (HRV) measures. These relationships should provide further information about interactions among the system’s constituents represented in terms of choir members’ individual arousal or sympathetic–parasympathetic balance and their HFN topology, which represents the structure of the superordinate system (choir) itself.

HRV is a useful tool for the detection of the sympathetic–parasympathetic balance of an individuals’ ANS and can be assessed by time- and frequency-domain analyses. For time-domain analysis, *SDNN* (standard deviation of interbeat intervals), and *RMSSD* (root mean square of successive differences) are the most commonly used measures. For frequency-domain analysis, the power spectral density of HRV signals is calculated, which is mostly divided into two frequency ranges: LF (low frequency: 0.04–0.15 Hz) and HF (high frequency: 0.15–0.40 Hz). These frequency ranges are related to the sympathetic and parasympathetic (or vagal) branches of the ANS. LF HRV may reflect both sympathetic and parasympathetic activity, while HF HRV primarily represents parasympathetic autonomic activity. Normally, the *LF/HF* ratio is used, representing the sympathetic–parasympathetic balance in ANS activity (Malik et al., 1996). Data on *HR* and HRV during singing are overall scarce and inconsistent (Bettermann et al., 2002; Grape et al., 2003; Cysarz et al., 2004; Harmat and Theorell, 2010; Marti, 2012; Olsson et al., 2013). Marti (2012) showed a significant decrease in *HR* and HRV as measured by *SDNN*, *pNN50* (i.e., the proportion of pairs of successive interbeat intervals that differ by more than 50 ms divided by the total number of interbeat intervals), and *RMSSD*, and a significant increase of the sympathovagal balance measured by *LF/HF* ratio during choral singing as compared to resting state. In a study with amateur singers comparing singing with slow breathing conditions (Olsson et al., 2013), *HR* was generally higher and HRV (measured by *SDNN* as well as LF and HF spectral power) was generally lower during singing than in slow breathing conditions. In another study (Grape et al., 2003), professional singers showed an increase in both the LF and HF spectral power of HRV (but not in *LF/HF* ratio) during singing in comparison to amateur singers. Vickhoff et al. (2013) investigated the influence of music structure on singing and found that hymn and mantra singing induced significantly higher *RMSSD* than a humming or baseline condition did, while the *LF/HF* ratio could only distinguish hymn singing from the other three conditions. They also found that music (singing) structure determines HRV and respiration rates with a clear tendency toward an entrainment between singers’ respiration and HRV signals.

In the present analysis, we expected the network topology of the choir to be distinguished by high *CC* and short *CPL* as well as high *E_local_* and *E_global_* during canon singing in parts as compared to canon singing in unison. Moreover, we aimed to compare the network topology calculated within canon groups with that calculated within randomly constructed groups of singers. The expectation was that above all, *CC* (and also *E_local_*) would be higher in the natural or real groups than in the randomly constructed groups. That is, the probability of neighbor–neighbor connections should prove higher in the former than in the latter. Correspondingly, we also expected *CPL* to be shorter and *E_global_* to be greater in real as compared to randomly constructed groups. Moreover, we assumed that the network topology in canon groups would be self-similar to that of the superordinate system itself. In addition, we hypothesized that network topology measures would be significantly related to individual *HR* and HRV measures indicating arousal and sympathovagal balance of the ANS. These correlations should provide the opportunity to better understand the network’s contribution to the singers’ physiological states. For this study, we re-analyzed the data collected from an amateur choir as presented in earlier works (Müller and Lindenberger, 2011; Müller et al., 2018a).

## Materials and Methods

### Participants

As described in depth previously (Müller et al., 2018a), five men and seven women (age range = 23.06–56.68 years; *M* = 35.7; *SD* = 10.69) were recruited from the choir of the Max Planck Institute for Human Development in Berlin, Germany. The group consisted of a conductor and eleven singers. Two of the twelve participants had professional musical training. Except for one person, all participants were able to play at least one instrument. The study was approved by the ethics committee of the Max Planck Institute for Human Development (Berlin), and performed in accordance with the ethical standards laid down in the 1964 Declaration of Helsinki. All subjects volunteered for this experiment and gave their written informed consent prior to their inclusion in the study.

### Procedure

After providing written informed consent and completing a questionnaire about their demographics and musical experience, the participants were connected to the measuring instruments. The participants were aligned in a predetermined position with the eleven singers facing the conductor and standing in two rows. The testing session began and ended with a relaxation condition. The canon “Signor Abbate” in B major (by Ludwig van Beethoven) was performed in three experimental variations: (i) singing the canon in unison (C_un_), (ii) singing the canon with three individual parts at regular intervals with eyes open (C_eo_), and (iii) singing the canon with three individual parts at regular intervals with eyes closed (C_ec_, the conductor sang along in the third part). All recordings lasted five minutes per condition and the tasks were separated by three-minute breaks.

### Data Acquisition and Analysis

The electrocardiogram (ECG), respiratory movement, and vocal audio signals were recorded simultaneously from all participants during each of the three conditions. All signals from all participants were sampled simultaneously at a 5000 Hz sampling rate using BrainAmp ExG bipolar amplifiers and BrainVision Recorder software (Brain Products GmbH, Gilching, Germany). The anti-aliasing filter was set to 1000 Hz. A notch filter was used to suppress 50 Hz noise. Simultaneously with the electrophysiological measures, a video was recorded with BrainVision Video Recorder software.

**Figure 1:**
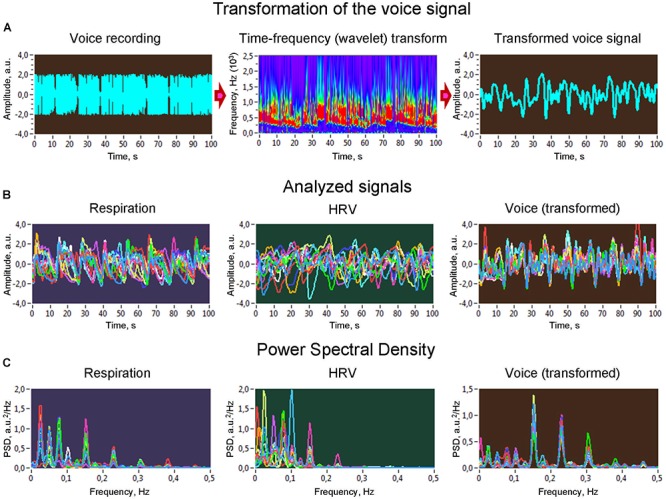
Transformation of voice signals and power spectral density of analyzed signals. (**A**) Raw signal of a voice recording (left), wavelet transform of the voice signal (middle), and new voice signal derived from the wavelet-transformed signal by averaging the wavelet power across the frequency bins (right). (**B**) Analyzed signals representing different subsystems investigated in the study: respiration, heart rate variability, and voice production. (**C**) Power spectral density of the analyzed signals. HRV, heart rate variability.

All signals analyzed in the study had a duration of 300 s. The QRS complexes in the ECG signals were used to identify beat locations. R peaks or beat locations were identified automatically using the BrainVision Analyzer program. Ectopic and other false beats were corrected manually. Once the timing of beats was determined, an instantaneous *HR* signal was created as a function of time, i.e., sampled with a given sampling rate. Vocal signals were adjusted to the low-frequency respiration and HRV time series using the time-frequency analytic wavelet transform with energy normalization and consecutive averaging of the spectral power within the frequency range of the maximal amplitude of frequency response (see Figure 1 and Müller et al., 2018a for details). This adjustment of high-frequency vocal signals takes into account slow modulations in the vocal signal, allowing us to operate in the same frequency range as in the case of respiratory and HRV signals. Thereafter, all signals were down-sampled to 4 Hz using the spline interpolation method. Before downsampling, the data were detrended and band-pass filtered between 0.002 and 4 Hz in order to eliminate linear trends and to remove very high frequency components. The Spencer’s 15-Point Moving Average method was used to smooth all time series in order to highlight the underlying structure. Furthermore, means were removed from the data and normalized to a unit variance. In the signals, the power spectral density was calculated using fast Fourier transform (FFT) with a Hamming window to determine the *LF/HF* ratio and spectral peaks for synchronization and network analyses.

To determine the physiological status quo of choir members, we calculated average *HR* of each individual during the three canon-singing conditions and also HRV measures such as *SDNN*, *RMSSD*, and *LF/HF* ratio. *SDNN* was determined by the formula:

$$SDNN\;=\;\sqrt{\frac{1}{n-1}\;\sum_{i=1}^{n}\;{\left(RR_{i}\;-\;\bar{RR}\right)}^{2}}\;\;\;\;\;\;\;\;\;\;\;\;\;\;\;\;\;\;\;\;\;\;\;\;\;(1)$$

and RMSSD by:

$$RMSSD\;=\;\sqrt{\frac{1}{n-1}\;\sum_{i=1}^{n-1}\;{\left(RR_{i+1}\;-\;RR_{i}\right)}^{2}}\;\;\;\;\;\;\;\;\;\;\;\;\;\;\;\;\;\;\;\;\;\;(2)$$

where *RR_i_* and *RR_i+1_* are consecutive interbeat intervals, RR is the mean interbeat interval, and *n* is the total number of interbeat intervals in the sequence. For calculation of the *LF/HF* ratio, LF and HF HRV were determined as a power spectral density in the frequency ranges 0.04–0.15 Hz and 0.15–0.40 Hz, respectively.

For the synchronization and following network analyses, ten different frequency components or frequencies of interest were chosen based on the spectral peaks in power spectrum of the signals under consideration and with regard to the fixed relation between the frequencies (1:2, 1:3, 2:3, etc.) being in line with the cardioventilatory phase synchronization approach reported by Mazzucco et al. (2017): 0.025, 0.050, 0.075, 0.100, 0.125, 0.150, 0.200, 0.250, 0.300, and 0.400 Hz. These frequency components comprise VLF, LF, and HF bands responsible for sympatho-vagal balance. To investigate phase synchronization within and between these frequencies, we applied an analytic complex-valued Morlet wavelet transform to compute the instantaneous phase in the frequency range from 0 to 2 Hz in steps of 0.0025 Hz. The wavelet coefficients were calculated with a time step of 2, leading to a time resolution of 0.5 s. WFC and CFC were determined using the integrative coupling index (*ICI*) algorithm (see Supplementary Material for details) described in our previous studies (Müller and Lindenberger, 2011; Müller et al., 2018a). The *ICI* is an asymmetric or directed coupling measure indicating both the common (absolute) and the “positive” or “leading” influence exerted by phase synchronization, and ranges between 0 and 1, with 0 indicating no synchronization and 1 indicating a fully synchronized time series.

### Network Construction and Network Properties

**Figure 2:**
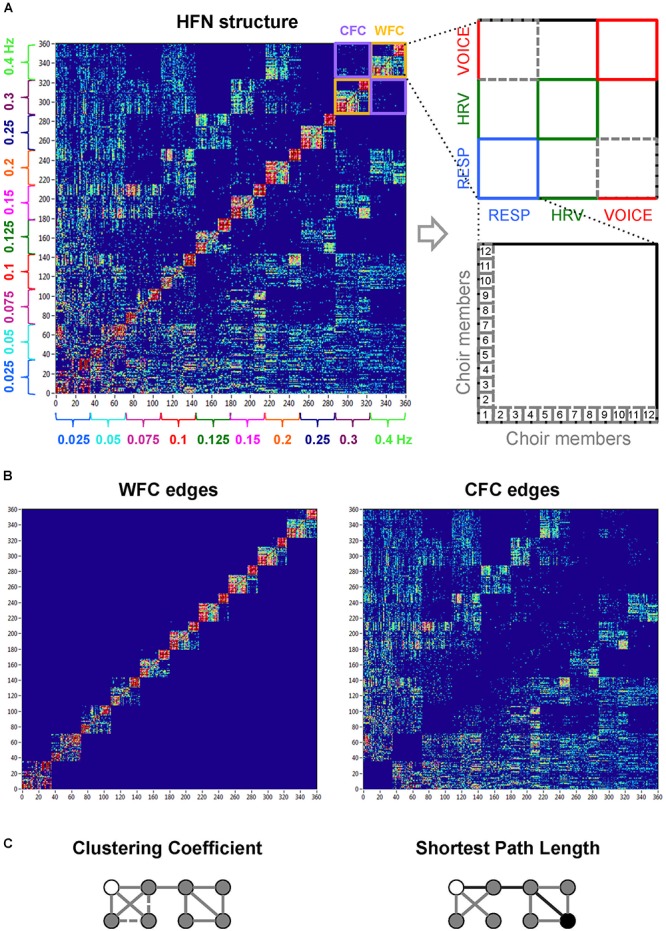
Hyper-frequency network representation of the choir. (**A**) Hyper-frequency network with 360 nodes (subsystems × frequency): each frequency contains 36 nodes (12 respiration + 12 HRV + 12 voice system nodes). The different subsystems on the right are depicted in different colors (respiration, blue; HRV, green; voice, red). (**B**) Representation of WFC (left) and CFC (right) edges within the hyper-frequency network. (**C**) Representation of network properties such as *CC* (left) and shortest path length (right). For simplicity, a binary undirected graph with 8 nodes is presented, where the existing links equal 1. The *CC* for the white node is 1/3; there is 1 neighbor–neighbor connection (solid line) by 3 possible connections (1 solid line + 2 dashed lines). The shortest path length between the white and the black node (depicted with black lines) is 3. *E_local_* is determined in a subgraph of a node *i*’s immediate neighbors, but not the node *i* itself; for the white node, it equals 1/3. *E_global_* is determined as the normalized inverse shortest path length between node *i* and all other nodes, and for the white node, it equals 0.643.

As before (Müller et al., 2018a), we constructed the network using WFC and CFC between all choir members determined for the three subsystems (HRV, respiration, and voice) oscillating at ten different frequencies each time. Please note that in contrast to our previous study (Müller et al., 2018a), the choir conductor’s hand movements were excluded from the analyses, so that all participants (including the conductor) have the same number of nodes. Thus, each choir member evinced 30 nodes (3 subsystems × 10 frequencies), and the common network contained 360 nodes altogether, covering all possible interactions between the choir members and including interactions within and between the subsystems and oscillation frequencies (see Figure 2). In order to determine the network properties, we set the cost level (ratio of the number of actual connections divided by the maximum possible number of connections in the network) to 25%, which makes it possible to investigate sparse networks. The connectivity threshold was always higher (*ICI* = 0.23) than the significance level determined by the surrogate data procedure (i.e., networks at this cost or sparsity level always included significant connections and had relatively similar number of edges). This allowed more accurate examination of the network topology in the different canon conditions. For further details regarding the network construction method and other information, please see also Müller et al. (2018a).

All network topology measures described below were first determined for each node separately, and then averaged for each choir member for statistical evaluation. This means that *m* in all the formulas above was equal to 30 (3 subsystems × 10 frequencies) corresponding to the number of nodes in each participant.

#### Degrees and Strengths

As *ICI* is a directed measure, we obtained the node in- and out-strengths as weighted in- and out-degrees. In-strength (*In-S*) is the sum of all weighted incoming connections, *S_in_* = ∑ _*j*∈*N*_ *w_ji_*, and out-strength (*Out-S*) is the sum of all weighted outgoing connections, *S_out_* = ∑ _*j*∈*N*_ *w_ij_*, where *w_ji_* are weights going from each node *j* to all other nodes *i* in the network and *w_ij_* are weights going from each node *i* to all other nodes *j* in the network. For statistical analyses, the strengths were averaged for each choir member across all nodes within the participant, including either all connections (WFC and CFC), or WFC and CFC connections separately (see Figure 2 for details).

#### Clustering Coefficient and Characteristic Path Length

For an individual node, the *CC* is defined as the proportion of the number of existing neighbor–neighbor connections to the total number of possible connections within its neighborhood. In the case of a weighted directed graph, the individual mean *CC* is calculated as follows (Fagiolo, 2007):

$$CC\;=\;\frac{1}{m}\;\sum_{i\in N}\;CC_{i}^{wd}\;=\;\;\frac{1}{m}\;\sum_{i\in N}\;\frac{t_{i}^{wd}}{\left(k_{i}^{out}\;+\;k_{i}^{in}\right)\;\left(k_{i}^{out}\;+\;k_{i}^{in}\;-\;1\right)\;-\;2\;\sum_{j\in N}a_{ij}a_{ji}}\;\;\;\;\;\;\;\;\;\;\;\;\;\;\;\;\;\;\;\;\;\;\;\;\;\;(3)$$

with 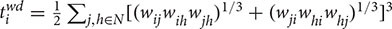 being the number of weighted directed triangles around a node *i*, 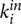 and 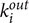 are in- and out-degrees of the node *i*, *a_ji_* and *a_ij_* are directed links of the adjacency matrix, and *m* is the number of nodes within the participant, *m* = 30. The *CC* measures the cliquishness of a typical neighborhood and is thus a measure of network segregation.

The shortest path length or distance *d_i,j_* between two nodes *i* and *j* is normally defined as the minimal number of edges that have to be passed to go from *i* to *j*. As our networks are directed weighted graphs, the weight and direction of the links must be considered. The input matrix is then a mapping from weight to length (i.e., a weight inversion), and the distance 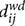 is the minimal weighted directed distance between the nodes *i* and *j*, but not necessarily the minimal number of edges. To calculate the *CPL* of an individual, path lengths between all possible pairs of vertices or nodes in the network were determined (Watts and Strogatz, 1998) and then averaged among nodes belonging to each specific individual:

$$CPL\;=\;\frac{1}{m}\;\sum_{i\in N}\;L_{i}^{wd}\;=\;\frac{1}{m}\;\sum_{i\in N}\;\frac{\sum_{j\in N,j\neq i}d_{ij}^{wd}}{n\;-\;1}\;\;\;\;\;\;\;\;\;\;\;\;\;\;\;\;\;\;\;(4)$$

whereby 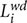 is the shortest path length of a node *i*, *n* is the total number of nodes in the network, and *m* is the number of nodes within the participant. *CPL* shows the degree of network integration with regard to an individual, with a short *CPL* indicating higher individual network integration.

### Global and Local Efficiency

Global efficiency (*E_global_*) is defined as the average inverse shortest path length. To determine the individual *E_global_*, we first calculated the so-called nodal efficiency for individual nodes and then calculated the average *E_global_* (Latora and Marchiori, 2001) for each participant:

$$E_{global}\;=\;\frac{1}{m}\sum_{i\in N}E_{global(i)}^{wd}\;=\;\frac{1}{m}\;\sum_{i\in N}\;\frac{\sum_{j\in N,j\neq i}(d_{ij}^{wd})-1}{n\;-\;1}\;\;\;\;\;\;\;\;\;\;\;\;\;\;\;\;\;\;\;\;\;\;\;\;\;(5)$$

whereby 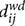 is the minimal weighted directed distance between the nodes *i* and *j*, *n* is the total number of nodes in the network, and *m* is the number of nodes within the participant. The nodal efficiency is practically the normalized sum of the reciprocal of the shortest path lengths or distances from a given node to all other nodes in the network, and individual *E_global_* quantifies how well a given participant is integrated within the network.

Local efficiency *(E_local_)* is similar to the *CC* and is calculated as the harmonic mean of neighbor–neighbor distances (Latora and Marchiori, 2001) also for each choir participant:

$$E_{local}\;=\;\frac{1}{m}\;\sum_{i\in N}\;E_{local(i)}^{wd}=\;\frac{1}{m}\;\sum_{i\in N}\;\frac{t_{e}^{wd}}{\left(k_{i}^{out}\;+\;k_{i}^{in}\right)\;\left(k_{i}^{out}\;+\;k_{i}^{in}\;-\;1\right)\;-\;2\;\sum_{j\in N}a_{ij}a_{ji}}\;\;\;\;\;\;\;\;\;\;\;\;(6)$$

with

$$t_{e}^{wd}\;=\;\frac{1}{2}\;\sum_{j,h\in N,j\neq i}\;(w_{ij}^{1/3}\;+\;w_{ji}^{1/3})(w_{ih}^{1/3}\;+\;w_{hi}^{1/3})\;({\left({\left[d_{jh}^{wd}(N_{i})\right]}^{-1}\right)}^{1/3}\;+\;{\left({\left[d_{hj}^{wd}(N_{i})\right]}^{-1}\right)}^{1/3}),$$

where *N_i_* denotes the subgraph comprising all nodes that are immediate neighbors of the node *i*, 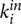, and 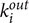 are in- and out-degrees of the node *i*, *a_ji_*, and *a_ij_* are directed links of the adjacency matrix, and *m* is the number of nodes within the participant. Thus, *E_local_* of node *i* is defined with respect to the subgraph comprising all of *i*’s neighbors, after removal of node i and its incident edges (Latora and Marchiori, 2001). Like *CC*, *E_local_* is a measure of the segregation of a network, indicating efficiency of information transfer in the immediate neighborhood of each node.

To compare network topology within the canon groups with randomly constructed groups, we calculated all the topology measures for three smaller groups, each consisting of four choir members singing a specific canon part. These subnetworks consist of 120 nodes comprising 4 choir members with 3 subsystems (i.e., respiration, cardiac, and voice) and 10 frequency components each (4 × 3 × 10 = 120). The randomly constructed networks had the same network structure, but the four choir members were chosen randomly (i.e., they were very likely to have sung different parts of the canon at the same time). This randomization procedure was repeated 100 times, and network topology measures were determined and averaged thereafter for statistical evaluation. It should be noted here that in the C_eo_ condition, the conductor did not sing within the third group throughout as she did in the C_ec_ condition, but participated in the different groups. In the C_un_ condition, real and random groups did not differ regarding their singing, because all singers sang the same part, but the members of the real groups were standing next to each other in the choir (i.e., in each other’s direct vicinity) and had previously sung the same part of the canon together in the C_eo_ and C_ec_ condition.

### Statistical Analysis

In- and out-strengths were determined for the whole network and separately for WFC and CFC. The remaining network metrics (i.e., *CC*, *CPL*, *E_local_*, and *E_global_*) were determined for the entire network only; they were first determined for each node separately and then aggregated and averaged for each subject. The network metrics as well as HR/HRV measures were then subjected to a one-way repeated measures ANOVA with the within-subject factor Condition (three different canon singing conditions: C_un_, C_eo_, and C_ec_). For statistical evaluation of the network topology determined within the real canon groups and within the randomly constructed groups, we conducted a two-way repeated measures ANOVA with the within-subject factors Grouping (real vs. random) and Condition (C_un_, C_eo_, and C_ec_). When necessary, Greenhouse-Geisser epsilons were used for non-sphericity correction in all ANOVAs. The Scheffé test was employed for the *post hoc* testing of condition differences. Real and random network topology measures within the different conditions were tested using a paired *t*-test.

## Results

### Network Metrics and Heart Rate Variability Measures

**Figure 3:**
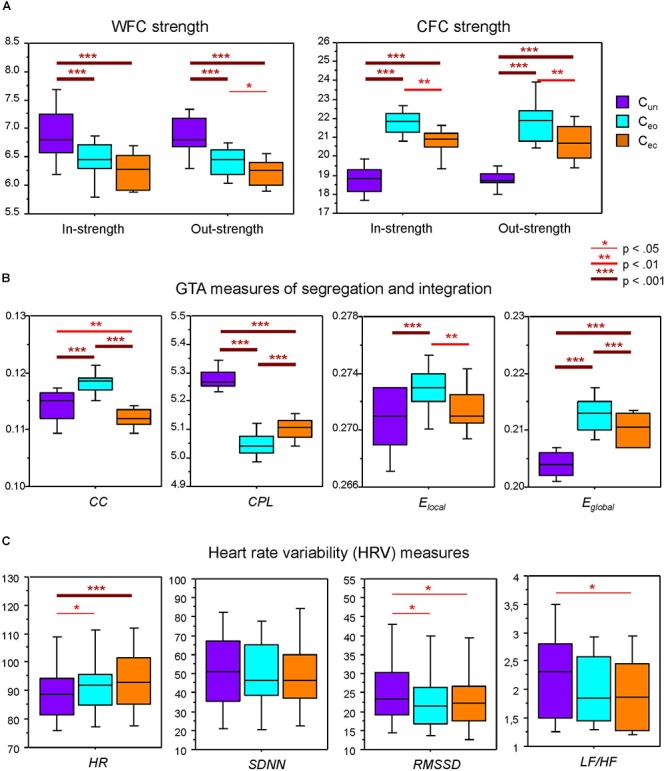
Boxplots of the network topology and HR/HRV measures under the three different canon conditions. (**A**) Within-frequency coupling (WFC) and cross-frequency coupling (CFC) in- and out-strengths under the three canon conditions: C_un_, canon singing in unison; C_eo_, canon singing with eyes open; and C_ec_, canon singing with eyes closed. (**B**) Segregation and integration graph-theoretical approach (GTA) measures (*CC*, clustering coefficient; *CPL*, characteristic path length; *E_local_*, local efficiency; and *E_global_*, global efficiency) under the three canon conditions (see legend in **A**). (**C**) Average heart rate (*HR)* and heart rate variability (HRV) measures (*SDNN*, *RMSSD*, and *LF/HF* ratio) under the three canon conditions (see legend in **A**). Asterisks indicate significant condition differences in the Scheffé test (^∗^*p* < 0.05, ^∗∗^*p* < 0.01, and ^∗∗∗^*p* < 0.001).

In- and out-strengths showed significant differences between conditions (see Table 1 for ANOVA results) and were generally significantly highest during canon singing with eyes open, and lowest when singing the canon in unison, as indicated by Scheffé *post hoc* tests. Separate analyses of WFC and CFC strengths showed such a relationship only for the CFC strengths but not for the WFC strengths, which were highest during C_un_ and lowest during C_ec_ (see Figure 3A). As shown in Figure 3B, *CC* was highest during C_eo_ and lowest during C_ec_. *E_local_* showed a similar relationship and was greatest during C_eo_. *CPL* was longest during C_un_ and shortest during C_eo_. In contrast, *E_global_* was highest during C_eo_ and lowest during C_un_.

**Table 1:** Statistical analysis (ANOVA) results for GTA and HR/HRV measures comparing canon singing in unison, canon singing in three parts (eyes open), and canon singing in three parts (eyes closed).

	df	*F*-value	*P*-value	η^2^
**GTA measures**				
*In-S*	2,22	39.43	<0.0001	0.78
*Out-S*	2,22	26.84	<0.0001	0.71
*In-S* (WFC)	2,22	16.37	<0.0001	0.60
*Out-S* (WFC)	2,22	34.01	<0.0001	0.76
*In-S* (CFC)	2,22	56.90	<0.0001	0.93
*Out-S* (CFC)	2,22	44.65	<0.0001	0.80
*CC*	2,22	52.61	<0.0001	0.83
*CPL*	2,22	155.98	<0.0001	0.93
*E_local_*	2,22	13.94	<0.0001	0.56
*E_global_*	2,22	75.19	<0.0001	0.87
**HR/HRV measures**				
*HR*	2,22	8.15	0.004	0.43
*SDNN*	2,22	0.49	0.46	0.04
*RMSSD*	2,22	3.73	0.057	0.25
*LF/HF*	2,22	3.27	0.075	0.23

*GTA, graph-theoretical approach; In-S, in-strength; Out-S, out-strength; CFC, cross-frequency coupling; WFC, within-frequency coupling; CC, clustering coefficient; CPL, characteristic path length; E_*local*_, local efficiency; E_*global*_, global efficiency; HR, heart rate; HRV, heart rate variability; SDNN, standard deviation of interbeat intervals; RMSSD, root mean square of successive differences; LF/HF, low frequency/high frequency ratio*.

To assess the physiological status of the choir singers, we evaluated average *HR* and three prominent HRV measures (i.e., *SDNN*, *RMSSD*, and *LF/HF*). A one-way repeated measures ANOVA showed significant differences between the canon conditions for *HR* and approximately significant differences for *RMSSD* and *LF/HF* ratio. *HR* was lower during C_un_ as compared with C_eo_ and C_ec_, whereas *RMSSD* was higher during C_un_ as compared with C_eo_ and C_ec_. *LF/HF* showed the same trend as *RMSSD* with significant differences between C_un_ and C_ec_ only (see Figure 3C and Table 1 for details).

**Figure 4:**
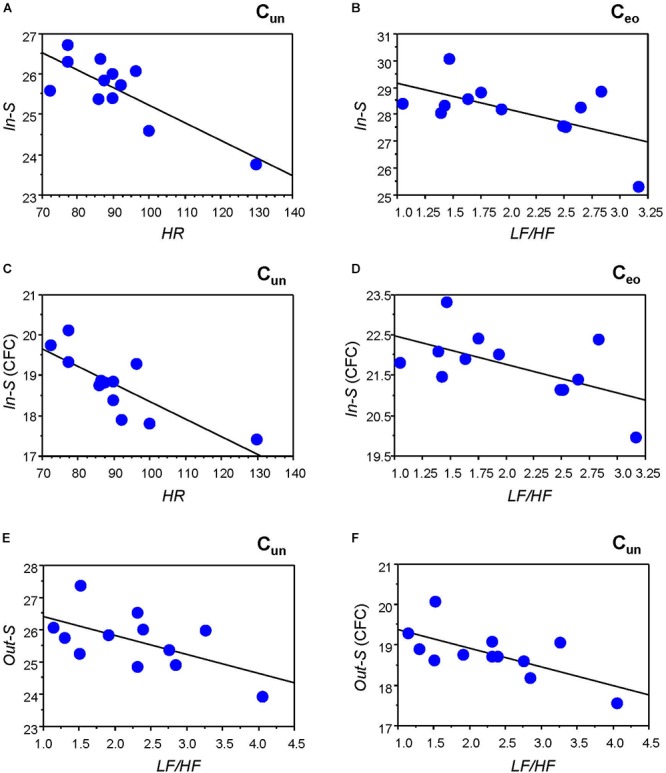
Correlation plots between strength and heart rate (*HR*) and heart rate variability (HRV) measures. (**A**) Correlation between *HR* and in-strength (*In-S)* while singing the canon in unison (C_un_). (**B**) Correlation between *LF/HF* ratio and *In-S* while singing the canon with eyes open (C_eo_). (**C**) Correlation between *HR* and *In-S* of CFC connections while singing the canon in unison (C_un_). (**D**) Correlation between *LF/HF* ratio and *In-S* of CFC connections while singing the canon with eyes open (C_eo_). (**E**) Correlation between *LF/HF* ratio and out-strength (*Out-S)* while singing the canon in unison (C_un_). (**F**) Correlation between *LF/HF* ratio and *Out-S* of CFC connections while singing the canon in unison (C_un_).

To determine the relationship between the network topology indicators and the physiological states of the individuals, we correlated the GTA metrics with *HR* and HRV measures. Significant correlations were found only for *HR* and *LF/HF*, and only during C_un_ and C_eo_ conditions. Figure 4 and Table 2 present some of these correlations. Both *HR* and *LF/HF* showed primarily negative correlations with GTA metrics (with the exception of *CPL*, which indicates a positive relationship with *HR* and *LF/HF* measures). Interestingly, significant correlations found for in- and out-strengths are due to CFC connections. Furthermore, the *LF/HF* ratio correlates significantly negatively with in-strengths during C_eo_ and with out-strengths during C_un_.

**Table 2:** Correlation between autonomic responses (*HR* and *LF/HF*) and choir network properties when singing a canon in unison (C_un_) or singing it in parts with eyes open (C_eo_).

GTA metrics	*HR*				*LF/HF*			
	C_un_		C_eo_		C_un_		C_eo_	
	*r*	*P*-value	*r*	*P*-value	*r*	*P*-value	*r*	*P*-value
*In-S*	**-0.799**	**0.001**	-0.502	0.098	-0.172	0.602	**-0.587**	**0.043**
*Out-S*	-0.547	0.066	-0.115	0.729	**-0.578**	**0.048**	-0.352	0.270
*In-S* (WFC)	-0.011	0.975	-0.477	0.120	0.085	0.798	-0.408	0.194
*Out-S* (WFC)	**-0.663**	**0.017**	-0.162	0.623	-0.274	0.399	-0.387	0.221
*In-S* (CFC)	**-0.801**	**0.001**	-0.428	0.170	-0.234	0.475	**-0.576**	**0.049**
*Out-S* (CFC)	-0.379	0.232	-0.096	0.772	**-0.667**	**0.016**	-0.319	0.322
*CC*	-0.489	0.109	**-0.587**	**0.043**	-0.153	0.643	-0.358	0.261
*CPL*	**0.647**	**0.021**	0.323	0.315	**0.626**	**0.028**	0.487	0.111
*E_local_*	**-0.653**	**0.019**	**-0.629**	**0.026**	-0.381	0.229	-0.554	0.061
*E_global_*	**-0.627**	**0.027**	-0.424	0.175	-0.494	0.105	-0.543	0.068

*Significant correlations are signed in bold. In-S, in-strength; Out-S, out-strength; CFC, cross-frequency coupling; WFC, within-frequency coupling; CC, clustering coefficient; CPL, characteristic path length; E_*local*_, local efficiency; E_*global*_, global efficiency*.

### Network Metrics in Real and Randomly Constructed Canon Groups

**Figure 5:**
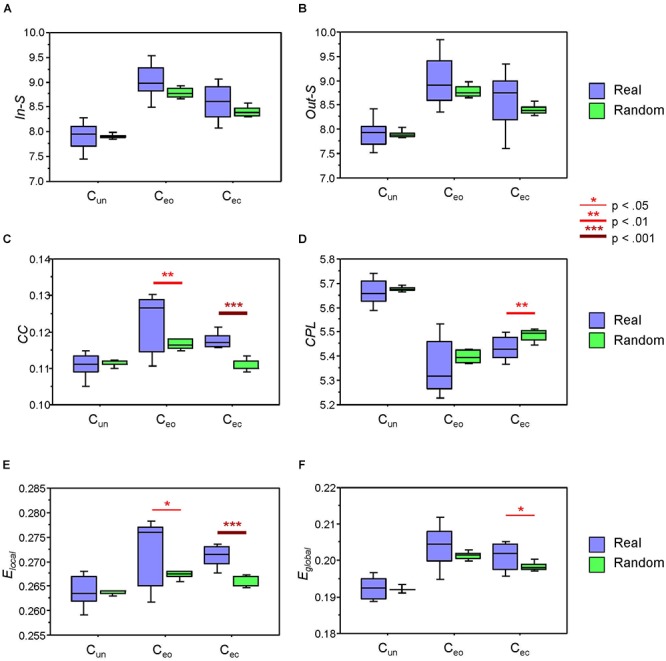
Boxplots of the network topology indices within the real and randomly constructed groups under the three different canon conditions: C_un_, canon singing in unison; C_eo_, canon singing with eyes open; and C_ec_, canon singing with eyes closed. (**A**) In-strengths (*In-S*). (**B**) Out-strengths (*Out-S*). (**C**) Clustering coefficient (*CC*). (**D**) Characteristic path length (*CPL*). (**E**) Local efficiency (*E_local_*). (**F**) Global efficiency (*E_global_*). Asterisks indicate significant grouping differences in the *t*-test (^∗^*p* < 0.05; ^∗∗^*p* < 0.01; and ^∗∗∗^ *p* < 0.001).

Next, we examined whether the network topology measures are different when calculated within the real canon groups and within randomly constructed groups. Results of these analyses are summarized in Figure 5 and Table 3. As shown, in- and out-strengths only revealed a significant main effect of Condition, whereas the other four GTA metrics or topology measures additionally yielded a significant main effect of Grouping, with greater *CC*, *E_local_*, and *E_global_* and shorter *CPL* in real canon groups as compared to randomly constructed groups. Although the interaction Grouping by Condition was significant only for *CC* and *E_local_*, there were no significant differences in the C_un_ condition but strong differences in the C_ec_ condition for all four measures, and also in the C_eo_ condition for *CC* and *E_local_* measures (see Figure 5 and Table 3 for details). As mentioned above, the conductor participated in different groups in the C_eo_ condition, whereas in the C_ec_ condition, she always sang with the same group. This appears to be the reason for stronger variation of the network topology measures in the C_eo_ condition. It can also be seen that network topology changes across conditions that were significant in all topology measures showed similar patterns as when calculated for the whole network. This indicates that condition-related differences are similar in the whole network as well as its parts.

**Table 3:** ANOVA results for network topology measures comparing real canon and randomly constructed groups (Grouping) singing a canon in unison, in parts with eyes open, or with eyes closed (Condition).

GTA measures/Factors	df	*F*-value	*P*-value	η^2^
***In-S***				
Grouping	1,11	3.84	0.076	0.26
Condition	2,22	72.96	<0.0001	0.87
Grouping × Condition	2,22	1.92	0.17	0.15
***Out-S***				
Grouping	1,11	1.88	0.20	0.15
Condition	2,22	48.82	<0.0001	0.82
Grouping × Condition	2,22	1.17	0.33	0.10
***CC***				
Grouping	1,11	19.54	0.0010	0.64
Condition	2,22	50.35	<0.0001	0.82
Grouping × Condition	2,22	14.01	0.0010	0.56
***CPL***				
Grouping	1,11	6.54	0.027	0.37
Condition	2,22	151.19	<0.0001	0.93
Grouping × Condition	2,22	1.38	0.27	0.11
***E_local_***				
Grouping	1,11	10.53	0.0078	0.49
Condition	2,22	44.43	<0.0001	0.80
Grouping × Condition	2,22	11.96	0.0020	0.52
***E_global_***				
Grouping	1,11	5.23	0.043	0.32
Condition	2,22	94.05	<0.0001	0.90
Grouping × Condition	2,22	2.19	0.15	0.17

*In-S, in-strength; Out-S, out-strength; CC, clustering coefficient; CPL, characteristic path length; E_*local*_, local efficiency; E_*global*_, global efficiency*.

## Discussion

To the best of our knowledge, network topology measures of singing in a choir have not been reported before (with the exception of network strength and modular organization of the choir network presented in our previous work; Müller and Lindenberger, 2011; Müller et al., 2018a). However, these measures have been extensively used in cognitive neuroscience to investigate brain network function and topology (for reviews, see Bassett and Khambhati, 2017; Liao et al., 2017), and also to examine hyper-brain (Sänger et al., 2012; Müller et al., 2013, 2018b; Müller and Lindenberger, 2014) and hyper-frequency (Müller and Lindenberger, 2014; Müller et al., 2016, 2018a) networks. Here, we examined the HFN topology of choral singing as indexed by cardiac, respiratory, and vocalizing activity expressed in ten different frequency components as a function of canon singing conditions. We also determined the physiological status quo of individuals singing in a choir using *HR* and different HRV measures and related them to the network topology measures to investigate the network’s contribution to the singers’ physiological states and vice versa. In addition, we determined HFN topology for real canon groups and compared it with that obtained from randomly constructed groups. We thereby provide information on a choir’s functioning as a superorganism or a superordinate system that imposes boundary conditions on its constituents.

We found that the HFN structure showed specific changes of network topology dependent on conditions of singing a canon. Overall strengths were higher when singing a canon in parts as compared to singing it in unison. However, separate analyses of WFC and CFC strengths showed that this relationship only applies to CFC strengths, while WFC strengths were strongest when singing the canon in unison (cf. Müller et al., 2018a). Moreover, other network metrics showed similar significant differences between canon conditions. Specifically, *CC*, *E_local_*, and *E_global_* were highest and *CPL* was, correspondingly, shortest during canon singing in parts with eyes open; in addition, *CPL* was longest and, correspondingly, *E_global_* lowest during canon singing in unison. This indicates that the choir network is more segregated and, at the same time, more integrated when singing the canon in parts. High segregation indicates that choir members build smaller clusters in the choir (e.g., canon groups singing different parts), while high integration can indicate that notwithstanding the high segregation of the choir, its members remain strongly connected to each other (e.g., attending to the singers in the other groups). The CFC connections apparently play a crucial role in this integration. High segregation and integration of the choir when singing the canon in parts also indicates that the choir HFN is a small-world network, especially in this condition (cf. Müller et al., 2018b). According to Watts and Strogatz (1998), small-world networks that are highly clustered and at the same time interconnected enhance signal-propagation speed, computational power, and synchronizability, and are unique in their ability to have specialized nodes exhibiting shared or distributed processing. This may explain why different canon groups are highly synchronized within the groups and are able to coordinate their actions between these groups at the same time. There is also evidence that behavioral coordination and synchronization may play a functional role in predicting others’ behavior and for successful joint action and communication (Sänger et al., 2011; Keller et al., 2014; Hasson and Frith, 2016; Koban et al., 2019). Predicting others’ behavior is also crucial for singing in a choir and must be linked to the network topology changes emerging during singing.

Furthermore, these metrics revealed a significant relationship to the *HR* as an indicator of arousal and to *LF/HF* ratio, an HRV index that reflects the balance between individuals’ sympathetic and parasympathetic activity. This suggests that network characteristics such as connectedness, segregation, and integration of the choir network are negatively related to the arousal and the sympathovagal balance of the ANS, at least in C_un_ and C_eo_ conditions. Interestingly, the *HR* was lowest and the HRV indices (i.e., *RMSSD* and *LF/HF*) highest during canon singing in unison. This indicates that singing the canon in unison was less arousing but required higher HRV or more sympathovagal balance of the ANS. There is evidence that an increase of *HR* and a decrease in HRV may reflect an increase of perceived stress (Dishman et al., 2000; Pollard et al., 2007; von Rosenberg et al., 2017). For example, Harmat and Theorell (2010) investigated *HR* and HRV changes in singers and flute players during rehearsal and concert, and found that the presence of an audience increased *HR* and reduced HRV. Moreover, they found that a high level of self-rated nervousness before the concert was associated with low LF power of HRV during a difficult piece in concert. Thus, it can be assumed that singing a canon in parts is more stressful than singing it in unison. Interestingly, as mentioned above, both the *HR* and HRV index (i.e., *LF/HF* ratio) correlated negatively with network topology metrics (a positive correlation with *CPL* indicates a correspondingly negative relationship with shorter path lengths). This means that lower *HR* and correspondingly lower stress are associated with stronger connectivity (above all in-strength), higher network integration, and segregation when singing canon in unison, whereas lower *HR* is only associated with higher network segregation when singing the canon in parts. Regarding HRV or *LF/HF* ratio, lower values (higher stress) are associated with higher out-strength (and also shorter *CPL*) in the unison condition and with higher in-strength when singing the canon in parts (C_eo_). In other words, stronger outgoing connections are more stressful when singing the canon in unison, and incoming connections are more stressful when singing the canon in parts. All this substantiates our suggestion that a choir network is not a rigid structure but rather a superorganism that changes its topology and interaction modes depending on the circumstances when singing in different conditions. Moreover, this structure, as expressed in different network topology indices, revealed relations to the singers’ ANS properties such as arousal and sympathovagal balance. The fact that different frequencies comprising LF and HF components contribute differently to network topology indicates that the sympathovagal balance during choir singing (also reflected in the *LF/HF* ratio) embodies a complex interplay among sympathetic and parasympathetic activities, which correspondingly regulate other subsystems (e.g., respiration and voice production) at the same or other frequencies. These findings are highly unlikely to be an epiphenomenon of a condition-invariant conductor-based clock generator but rather a property of the choir as a superordinate system in which the conductor plays a crucial role.

In our previous analyses (Müller and Lindenberger, 2011; Müller et al., 2018a), we were able to separate the canon parts only if we considered respiration alone or respiration together with voice signals oscillating at single (predominantly low) frequencies. In the present work, we considered all subsystems and all frequency components when comparing real canon groups with randomly constructed groups and found strong differences between the former and the latter, especially in the *CC* and *E_local_* topology measures. In other words, when determining the *CC* and *E_local_* within the real groups, the probability that two neighbors of a node are connected with each other is much higher than in the randomly constructed groups. Interestingly, *CPL* is shorter and *E_global_* correspondingly higher in the real groups than in the randomly constructed groups (at least in the C_ec_ condition, when the conductor always sang with the same group), indicating that the real groups are strongly integrated. Thus, HFN topology measures (especially *CC* and *E_local_*) are able to separate or at least distinguish different voices sung in a choir. High clustering and also *E_local_* within the groups accompanied by strong integration of all subsystems and frequency components indicate that choir members singing the same canon part possess intertwined and functionally effective network topology, although their overall connectedness (indicated by in- and out-strengths) does not rise and is comparable with that of randomly constructed groups. At the same time, the whole choir network exhibits high segregation and integration properties with high E_local_ and *E_global_* when singing a canon in parts as compared to singing it in unison. This indicates that both the superordinate system and its parts have sufficient network topology at their disposal, thereby allowing accurate and effective functioning under different circumstances. In this regard, they are self-similar.

The present study has some limitations and leaves room for questions to be addressed in future research. First, the sample size of our study was small with a wide age–gender distribution range. Further studies with larger samples and a smaller age–gender distribution range would provide more reliable information about coupling mechanisms during singing and would enhance the generalizability of the findings. Second, we examined a group of participants who were used to singing together regularly (albeit in an amateur choir), and our findings may not extend to spontaneous group singing, which is also known to engender happiness. Third, the *ICI* measure reflects only one specific type of synchronization, namely the in-phase synchronization or more specifically the common (absolute) and the “positive” or “leading” influence exerted by phase synchronization in defined phase angle boundaries. Other types of synchronization (e.g., non-linear coupling measures or other phase synchronization measures reflecting the entire range of phase differences) may provide additional information on the network processes.

Our results further extend previous work on the reach of network interactions during choral singing and highlight the way in which network topology supports cooperation and integration of temporally coordinated forms of social interaction. Future research could examine the reported effects of choral singing on well-being in this context (Stewart and Lonsdale, 2016). Perhaps becoming part of the superorganism that a choir constitutes has pleasurable effects that are dependent on certain aspects of the emerging network. In an electroencephalography hyperscanning study, it has been shown that hyper-brain HFNs emerging during romantic kissing are strongly related to partner-oriented kissing satisfaction and kissing quality (Müller and Lindenberger, 2014). This result is in line with our assumption about the pleasurable effects of singing and emerging complex networks linking the individuals in a choir.

We conclude that network topology dynamics provide crucial information about mechanisms of social interaction and may represent an efficient indicator for group social behavior and group dynamics. It supports a more general conjecture that network topology dynamics indexed by GTA measures may reflect and support interpersonal action coordination in other situations than singing, e.g., playing collective sports or music, dancing, mother–child interactions, and a wide range of social bonding behaviors.

## Data Availability

The datasets for this study will not be made publicly available because restrictions included in the consent statement that the participants of the study signed only allow the present data to be used for research purposes within the Max Planck Institute for Human Development in Berlin.

## Author Contributions

All authors designed the study, discussed the results, wrote the article, and read and approved the final version of the manuscript. VM acquired and analyzed the data.

## Conflict of Interest Statement

The authors declare that the research was conducted in the absence of any commercial or financial relationships that could be construed as a potential conflict of interest.

## Abbreviations

ANS: autonomic nervous system
*CC*: clustering coefficient
CFC: cross-frequency coupling
*CPL*: characteristic path length
*E_local_*: local efficiency
*E_global_*: global efficiency
GTA: graph-theoretical approach
HFN: hyper-frequency network
*ICI*: integrative coupling index
WFC: within-frequency coupling.
