# Supplementary material for: Hyper-Frequency Network Topology Changes During Choral Singing
Source: Front Physiol. 2019 Mar 6;10:207. doi: 10.3389/fphys.2019.00207 (PMC6416178; doi:10.3389/fphys.2019.00207)
Supplement: Supplementary file 1 [file Data_Sheet_1.PDF]

Supplementary Material

Calculation of *Integrative Coupling Index (ICI)*

To investigate phase coupling in a directed and frequency-resolved manner, we applied an analytic or complex-valued Morlet wavelet transform to compute the instantaneous phase in the frequency range from 0 to 2 Hz in 0.0025-Hz steps (see Fig. S1A). The complex mother Morlet wavelet, also called Gabor wavelet, has a Gaussian shape around its central frequency f:

$$w(t,f) = \left(\sigma^2\pi\right)^{-1/4} e^{\left(\left(-t^2/2\sigma^2\right)+3/2\pi jft\right)}, j = \sqrt{-1} \qquad (1)$$

in which σ is the standard deviation of the Gaussian envelope of the mother wavelet, with f and t denoting frequency and time bins, respectively. The wavelet coefficients were calculated with a time step of 2 leading to a time resolution of 0.5 s and frequency resolution of 0.0025 Hz. In order to identify the phase relations within and between any two channels or frequencies, the instantaneous phase difference was then computed from the wavelet coefficients for all possible electrode and frequency pairs (Fig. S1B). On the basis of instantaneous phases for two signals (X and Y) given as: $\Phi_X(f_m,t) = \arg[\phi_X(f_m,t)]$ and $\Phi_Y(f_n,t) = \arg[\phi_Y(f_n,t)]$, correspondingly, with ϕ_X and ϕ_Y being complex numbers, we determined the $n{:}m$ phase synchronization between two oscillations at the frequencies f_m and f_n. The generalized phase difference (ΔΦ) according to $n{\cdot}f_m = m{\cdot}f_n$ was calculated by:

$$\Delta\Phi\left(f_m,f_n,t\right) = n\cdot\Phi\left(f_m,t\right) - m\cdot\Phi\left(f_n,t\right), \bmod 2\pi \qquad (2)$$

In the case of WFC with $f_m = f_n$, the phase difference ΔΦ is calculated in the same way by setting $m = n = 1$.

We then calculated synchronization indices reflecting in-phase synchronization between two signals, that is, the extent to which the angle of phase differences approximates zero. Given the estimates of the phase difference between pairs of signals, it is possible to ascertain how long the phase difference remains stable in defined phase angle boundaries by counting the number of points that are phase-locked in a defined time window. We divided the range between −π/4

and $+\pi/4$ into two ranges and distinguished between positive and negative deviations from phase zero. As shown in Figure S1C, we marked negative deviations in the range between $-\pi/4$ and 0 in blue (coded as "−1") and positive deviations in the range between 0 and $+\pi/4$ in red (coded as "+1"). Phase difference values beyond these range were marked in green (coded as "0") and represent non-synchronization. In the case of two signals, S1 and S2, a blue stripe would mean that the phase of signal S2 precedes the phase of signal S1, and a red stripe would mean that the phase of signal S1 precedes the phase of signal S2. We then counted the number of data points that are phase-locked in each of these two ranges separately. Before counting, successive points in the defined range (between $-\pi/4$ and $+\pi/4$) with a time interval shorter than a period of the corresponding oscillation at the given frequency ($T_i = 1/f_i$) were discarded from the analysis. This cleaning procedure effectively eliminated instances of accidental synchronization. On the basis of this count, we obtained several synchronization indices: (1) the Positive Coupling Index, *PCI*, or the relative number of phase-locked points in the positive range (between 0 and $+\pi/4$); (2) the Negative Coupling Index, *NCI*, or the relative number of phase-locked points in the negative range (between $-\pi/4$ and 0); (3) the Absolute Coupling Index, *ACI*, or the relative number of phase-locked points in the positive and negative range (i.e., between $-\pi/4$ and $+\pi/4$) indicating in-phase synchronization; and (4) the Integrative Coupling Index, *ICI*, calculated by the formula (Müller & Lindenberger, 2011):

$$ICI = \frac{PCI + ACI}{2 \cdot ACI} \cdot \sqrt{PCI} \tag{4}$$

All these coupling measures are related to all measurement points in the window and range between 0 and 1. Note that the *ICI* is an asymmetric coupling index ($ICI_{AB} \neq ICI_{BA}$), indicating both the common (absolute) and the "positive" or "leading" influence exerted by phase synchronization in defined phase angle boundaries. In this article, we only report results on the *ICI* measure, which is the most informative due to its directionality.

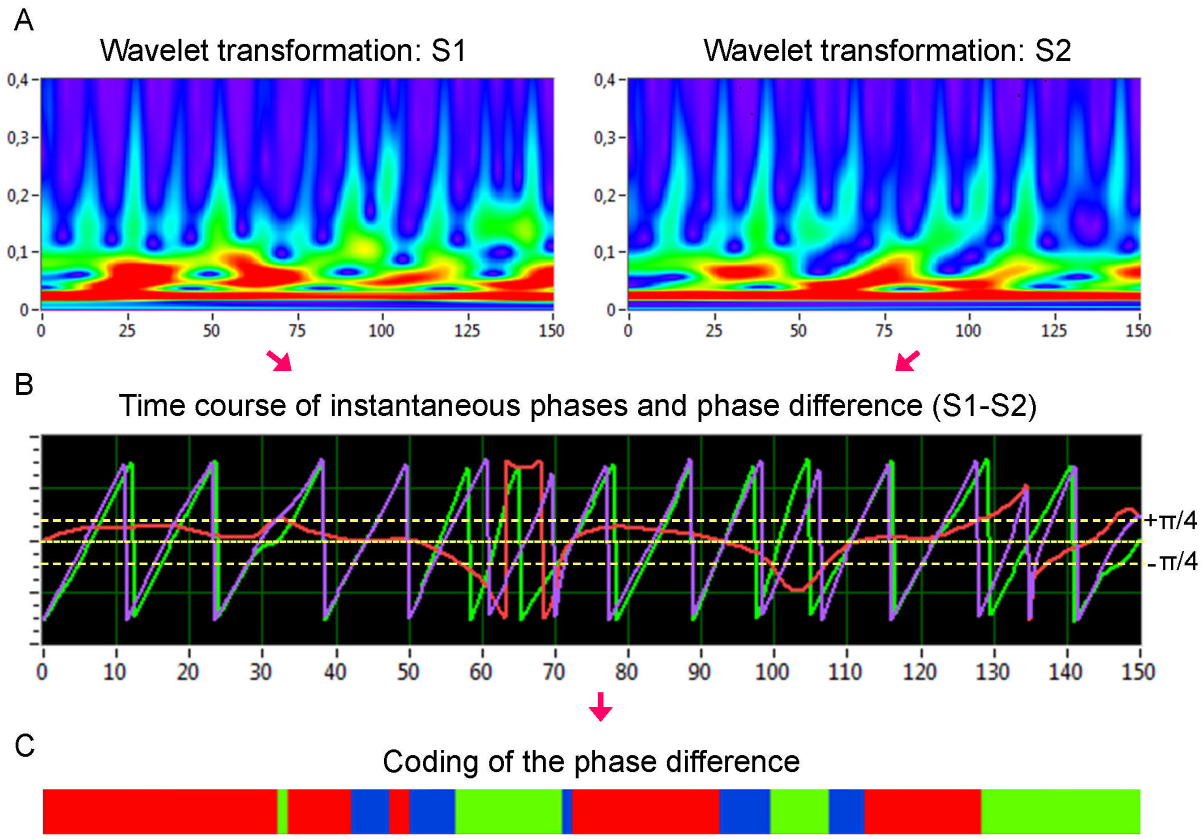

Number of red points (S1>S2) = 310; Number blue points (S1<S2) = 108; Total number of points = 600;
PCI = Number of red points/Total number of points = 310/600 = 0.517
NCI = Number of blue points/Total number of points = 108/600 = 0.180
ACI = Number of red and blue points/Total number of points = 418/600 = 0.697

$$ICI = \frac{PCI + ACI}{2 \cdot ACI} \cdot \sqrt{PCI} = \frac{0.517 + 0.697}{2 \times 0.697} \times \sqrt{0.517} = 0.626$$

Figure S1. Schematic presentation of the calculation of the Integrative Coupling Index (ICI). (A) Complex Morlet wavelet transformation of signals (S1 and S2) from two subjects in the time-frequency domain. (B) Time course of instantaneous phases from two signals and their phase difference (S1 = violet curve; S2 = green curve; S1–S2 = red curve). (C) Coding of the phase difference (-π/4 < S1–S2 < 0: blue stripes; 0 < S1–S2 < +π/4: red stripes; S1–S2 < -π/4 or S1–S2 > +π/4: green stripes = non-synchronization).
